# Severe Post-prandial Pain: A Case Report

**DOI:** 10.7759/cureus.22448

**Published:** 2022-02-21

**Authors:** Nandita Kakar, Harrison C Smith, David L Crawford, Anthony M Shadid

**Affiliations:** 1 Medicine, Nova Southeastern University Dr. Kiran C. Patel College of Osteopathic Medicine, Fort Lauderdale, USA; 2 Surgery, Springfield Clinic, Peoria, USA; 3 Radiology, University of Illinois at Chicago, Peoria, USA

**Keywords:** chronic abdominal pain, doppler ultrasound, median arcuate ligament release, celiac trunk, post prandial abdominal pain

## Abstract

Median arcuate ligament syndrome (MALS) is a rare cause of post-prandial abdominal pain due to compression of the celiac artery and celiac plexus. Associated symptoms include nausea, vomiting, diarrhea, and weight loss. The incidence of radiologic compression of the celiac axis is reported to be between 10% and 24%; however, symptomatic compression is noted to be found in about half of the population. MALS is considered a diagnosis of exclusion due to its tendency to present with nonspecific symptoms that mimic other common causes of abdominal pain. Radiologic evidence from angiography with breathing maneuvers is the gold standard for diagnosis. Surgical division of the median arcuate ligament to decompress the celiac artery is an effective treatment proving to provide up to 60-70% of symptomatic relief.

## Introduction

Abdominal pain accounts for 7-10% of emergency room visits, and approximately 25% of these patients are discharged with the diagnosis of undifferentiated abdominal pain [1].^ ^The differential for unspecific abdominal pain symptoms is broad and can require an extensive workup. We describe a case of a 36-year-old male who presented with syncope in the setting of a two-year-long recurring abdominal pain that worsened after meals. Previous episodes of the patient's post-prandial pain were attributed to gastroesophageal reflux and lifestyle; however, the persistence of symptoms led to further exploration and exclusion of many differential diagnoses. This case demonstrates the workup leading to the diagnosis of celiac artery compression by the median arcuate ligament.

## Case presentation

A 36-year-old Caucasian male with a past medical history of gastroesophageal reflux disease (GERD), celiac disease, diabetes, alcohol abuse, depression, and anxiety presented to the emergency department with severe abdominal pain resulting in syncope for an unknown duration. The patient was brought to the hospital after being discovered unconscious by a friend. Questioning revealed a two-year history of post-prandial, stabbing, mid-epigastric pain with occasional radiation to the right shoulder that had progressively worsened. The patient stated the pain was exacerbated by eating. He had been taking famotidine and omeprazole twice a day, which provided minimal relief. The patient also admitted to an unintentional weight loss of 10-12 pounds over the past year.

The patient was diagnosed with GERD and suspected peptic ulcer disease (PUD) in his late twenties. He previously underwent evaluation with upper and lower endoscopy, which did not reveal any significant findings. At that time, he was prescribed famotidine and omeprazole to treat his GERD symptoms, as well as dicyclomine and orphenadrine for intestinal spasms and cramping. Given the patient's history of non-compliance and substance abuse, further testing was not implemented and he was counseled on diet and lifestyle. 

On arrival, the patient was afebrile and hemodynamically stable. On physical examination, the patient was tall and thin, appearing anxious with an abnormal affect. He had mild pain to palpation in the epigastric region of his abdomen but was otherwise unremarkable. Comprehensive metabolic panel results revealed serum sodium of 133 mmol/L, potassium of 3.4 mmol/L, chloride of 99 mmol/L, blood urea nitrogen (BUN) of 16 mg/dL, creatinine of 1.59 mg/dL, and glucose of 311 mg/dL. Complete blood count results showed a white blood cell count of 8. 39 x 103/mcL, hemoglobin of 17.6 g/dL, hematocrit of 51.9%, and platelet count of 2.98 x 103/mcL. The urinalysis showed a specific gravity of 1.015, pH of 6.5, and 1+ glucosuria. The patient’s metabolic derangements were corrected with IV fluids, potassium, and insulin administration upon admission. Renal function was monitored daily throughout the admission.

A chest X-ray was obtained and revealed no abnormalities. Hepatobiliary scan findings were normal with no evidence of cholecystitis or biliary obstruction. CT of the abdomen and pelvis with IV contrast revealed a focal wedge-shaped hypodensity in the subcapsular aspect of the spleen, raising suspicion for vascular compromise. Further testing for a vascular etiology contributing to post-prandial pain was planned.

A mesenteric artery duplex scan and computed tomography angiography (CTA) were performed to evaluate the patient’s abdominal vasculature. The normal range of resting peak systolic velocity of the celiac artery is 98-105 cm/s and the patient's value was elevated, at 487 cm/s. Flow velocities within the splenic, hepatic, and superior mesenteric arteries were within normal limits. The inferior mesenteric artery (IMA) was shown to be patent. A repeat abdominal CT with contrast was performed two days after continuous supportive care to monitor the splenic hypodense lesion. There was no longer any evidence of the lesion and it seemed to have resolved. However, the patient's symptoms remained unchanged.

**Figure 1:**
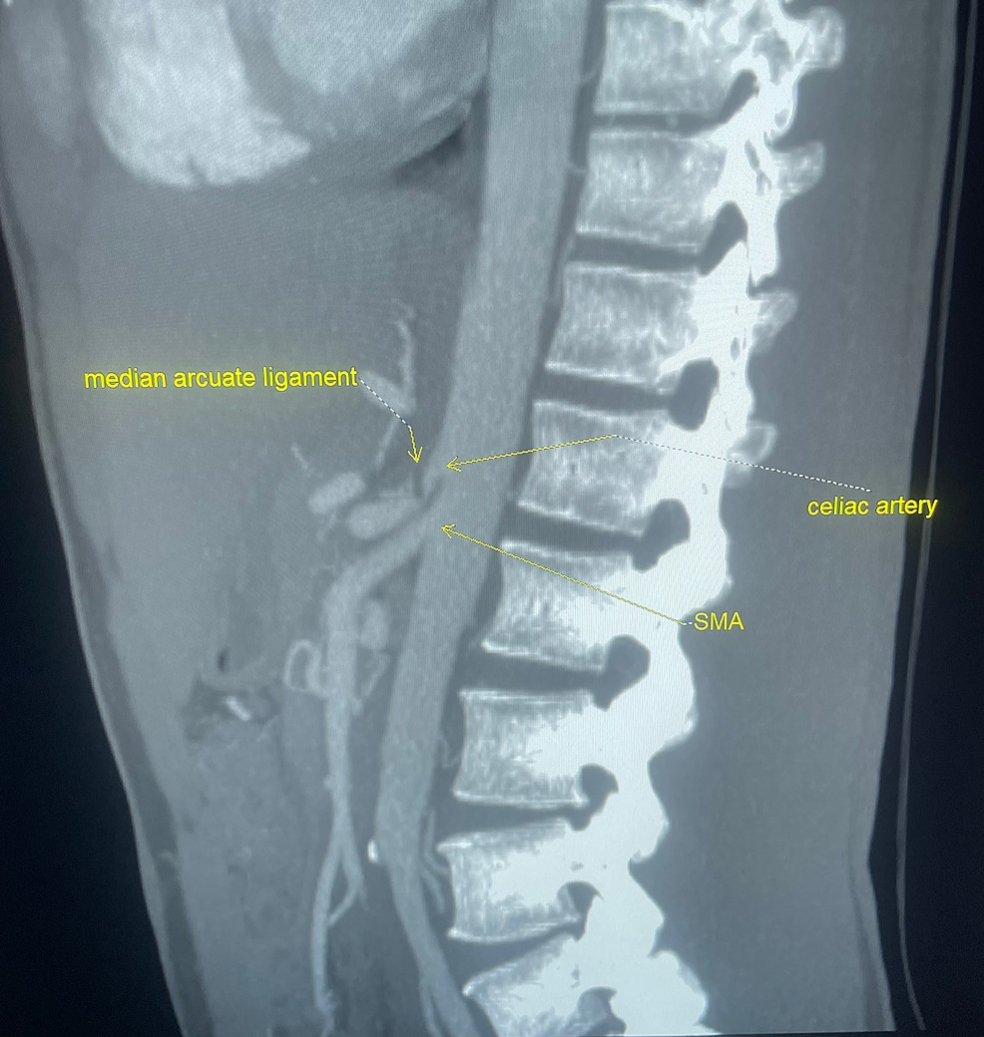
Computed tomography angiography showing celiac artery stenosis due to overlying median arcuate ligament with post-stenotic dilation. SMA = superior mesenteric artery.

Given the abnormally high velocity of the celiac artery, CTA of the celiac trunk was performed and displayed high-grade stenosis in the celiac artery with a band-like narrowing during the arterial phase, consistent with compression by the median arcuate ligament (Figure 1).

The patient received vascular surgery consultation for a robotic-assisted median arcuate ligament release procedure. The surgery was performed promptly without complication. At a two-week postoperative follow-up, the patient reported complete resolution of pain. Upon further follow-up, the patient only reported partial symptomatic relief. However, he reported a significant increase in quality of life suggesting a successful treatment outcome.

This case shows how symptoms caused by the compression of the celiac artery by the median arcuate ligament can likely be accompanied and further exacerbated by co-existing medical conditions. Surgical release may or may not provide a complete resolution and patients should continue following up with primary care to maintain hemodynamic and metabolic function for the potential of upgoing improvement.

## Discussion

Our patient presented with syncope after an episode of severe abdominal pain. The patient had previously undergone GI evaluation; however, the patient’s inadequate response to medical therapy led to further investigation with mesenteric duplex and CT angiography, resulting in a diagnosis of median arcuate ligament syndrome (MALS).

MALS is a rare medical condition with an incidence of two per 100,000 and is commonly seen in women between the ages of 30-50 years. MALS is characterized by the triad of recurrent, post-prandial abdominal pain, weight loss, and an abdominal bruit. The abdominal pain is caused by compression of the celiac artery by the median arcuate ligament, a fibrous band that connects the medial ends of the diaphragmatic crura. Variations in individual anatomy may contribute to this syndrome, such as an abnormally cephalad celiac trunk or caudad insertion of the diaphragm, which have been found to have a radiographic incidence of 10%-24% in some populations. During expiration, superior movement of the diaphragm causes the crura of the diaphragm to come into closer proximity to the celiac trunk, resulting in further compression [2].

The clinical presentation of MALS is highly variable and is often non-specific. The mechanism for pain in this syndrome is not completely understood. However, leading theories suggest that mesenteric ischemia and irritation of the celiac plexus are implicated. Post-prandial pain is believed to be due to mesenteric ischemia. After meals, oxygen demand is increased in gastrointestinal organs, and in patients with MALS, this demand cannot be met due to compression of the celiac trunk. In our patient, this phenomenon may have also contributed to the finding of splenic infarction, as the splenic artery is a branch of the celiac trunk. Referred pain from the spleen and diaphragm is known as Kehr’s sign and may be present and suggest splenic involvement. However, patients may be asymptomatic due to sufficient collateral blood supply [3]. The postprandial steal phenomenon describes retrograde flow from the superior mesenteric artery (SMA) through collateral vessels to branches of the celiac trunk, improving oxygen supply to celiac-supplied organs [4]. However, it is possible that this may result in inadequate oxygen supply to portions of the intestine supplied by the SMA. It has been suggested that adopting dietary habits that consist of smaller, more frequent meals will decrease intestinal blood flow and, consequently, the oxygen demand during digestion, possibly improving symptoms [3]. An alternate theory suggests the pain is due to chronic irritation and compression of the celiac plexus, a component of the sympathetic nervous system, causing pain and splanchnic vasoconstriction with resultant ischemia [5].

MALS is often a diagnosis of exclusion involving thorough workup to rule out disorders with similar presenting symptoms such as PUD or gallbladder disease.

Radiologic advancements have allowed for a shift from traditionally used catheter-angiography towards Doppler ultrasound and three-dimensional (3D) computed tomography imaging for confirming the diagnosis of MALS. The development of 3D reconstruction of CTA images allows clinicians to visualize the celiac artery and its surrounding anatomy. Focal narrowing of the celiac trunk, classically described as having a hooked appearance, is a characteristic finding and is best visualized in the sagittal plane [6]. Further supporting evidence includes visualizing the presence of collateral vessels, as well as the absence of significant atherosclerotic disease.

**Figure 2:**
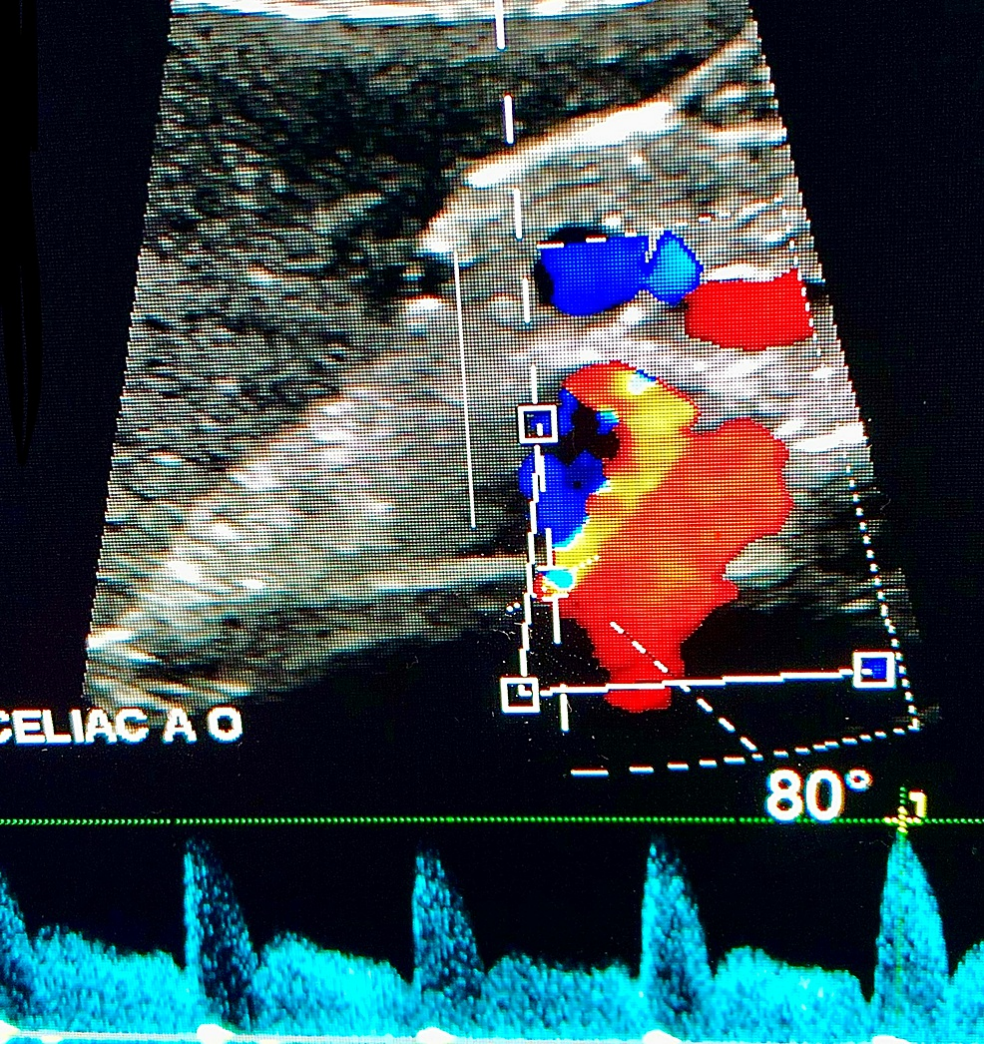
Mesenteric ultrasound showing the angle between the celiac artery and aorta on deep inspiration.

**Figure 3:**
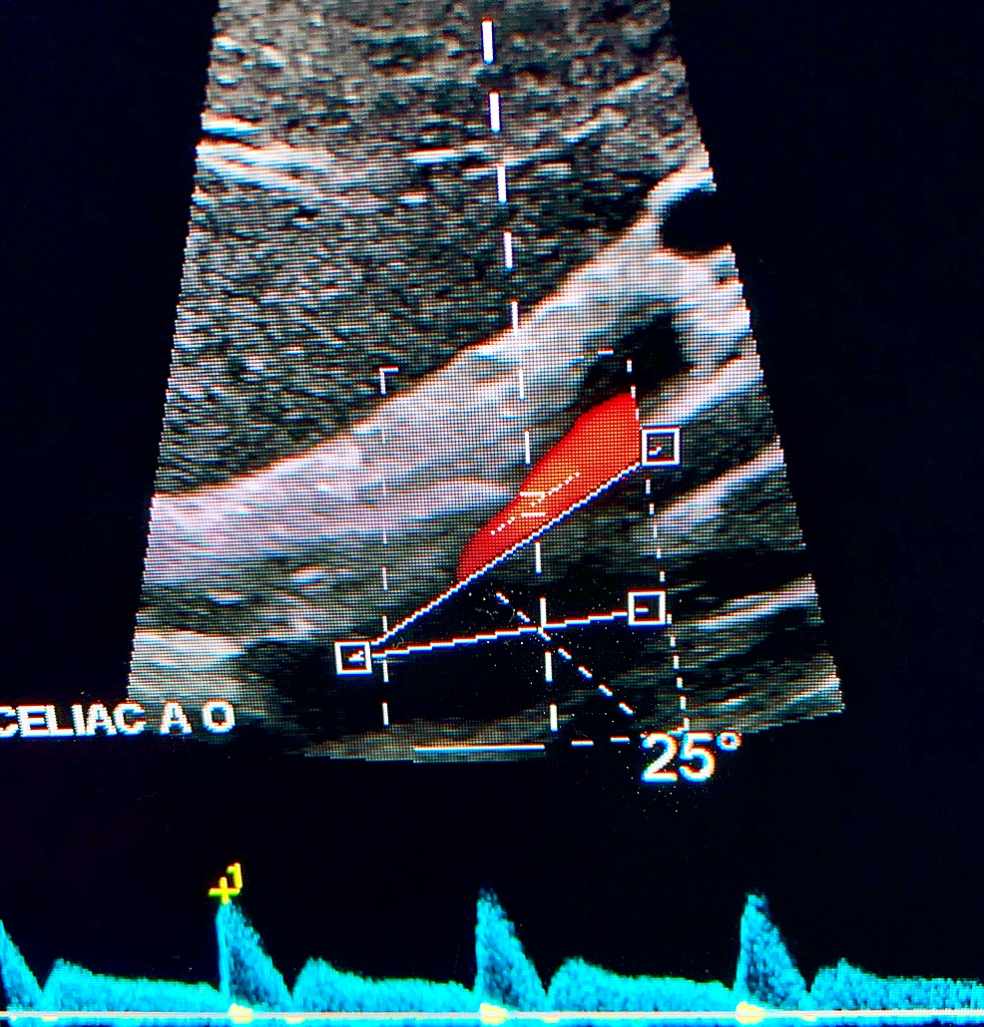
Mesenteric ultrasound showing the angle between the celiac artery and aorta on deep expiration.

Doppler ultrasound studies of the celiac artery may be performed to measure peak flow velocities of the celiac trunk. Measurements are recorded during both inspiration and expiration, as the degree of celiac artery stenosis is augmented by the respiratory cycle in MALS. During expiration, superior movement of the diaphragm exacerbates the degree of celiac artery stenosis, leading to elevations in peak expiratory flow velocities. The diagnostic criteria include at least one of the following: peak expiratory flow velocity greater than 350 cm/s in the celiac trunk or a deflection angle greater than 50° [7]. The angle between the aorta and the celiac artery is measured during inspiration and expiration. The deflection angle is the difference between these two values. In this case, the celiac artery was 80° from the aorta with a velocity of 240 cm/s on inspiration and 25° from the aorta with a velocity of 458 cm/s on expiration (Figures 2, 3). In this patient’s case, both peak expiratory flow velocity, as well as deflection angle, met the diagnostic criteria for MALS.

**Figure 4:**
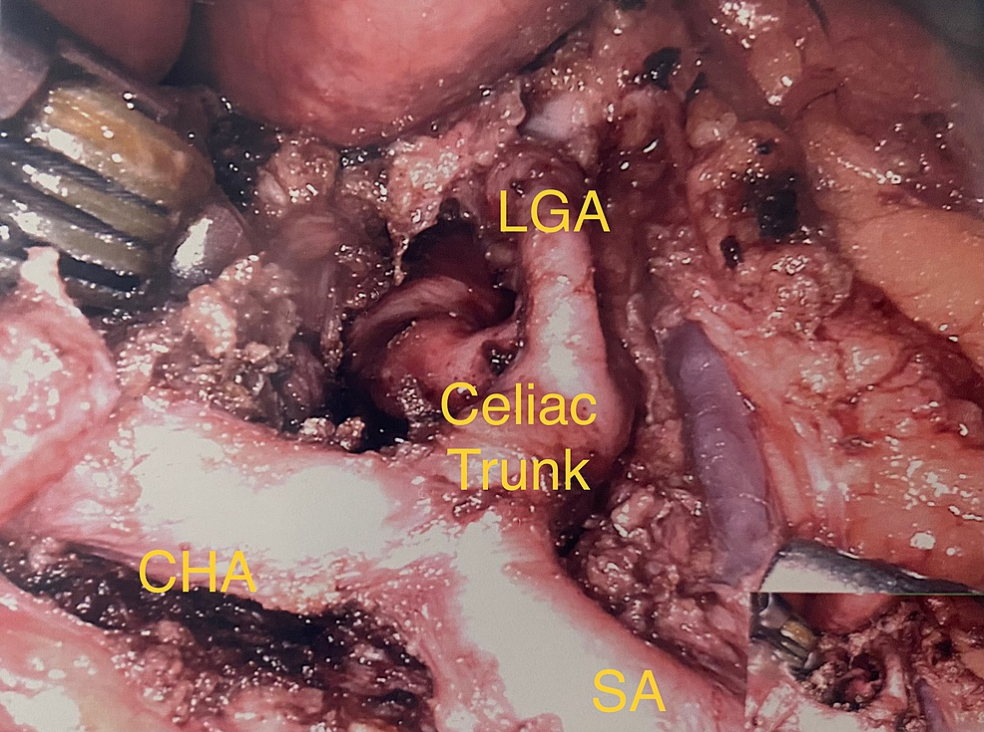
Post-decompression of the celiac trunk from overlying median arcuate ligament and celiac plexus fibers. CHA = common hepatic artery; LGA = left gastric artery; SA = splenic artery.

Surgical median arcuate ligament release is the treatment of choice. There is no known medical treatment for MALS. The patient in this case underwent robotic decompression of the celiac trunk with combined neurolysis of the celiac nerve plexus (Figure 4). The latter may improve symptom relief due to differing theories behind the etiology of this syndrome [8]. Other surgical approaches such as open or laparoscopic may be considered in certain cases depending on the characteristics and desires of the patient. Surgical treatment has resulted in partial symptomatic relief in many patients in up to 56% of patients, completely resolved symptoms in up to 37% of patients, and no relief in the remaining 7% [9].

## Conclusions

This case shows the common misdiagnosis of MALS due to its nonspecific symptoms, which mimic other common causes of abdominal pain. MALS should be considered a differential diagnosis for patients, especially young and thin females with ambiguous post-prandial abdominal pain when other etiologies have been ruled out. Diagnostic workup should include mesenteric ultrasound to measure arterial velocities and the deflection angle. Abnormally high flow velocity indicates significant stenosis, which can be confirmed by CT angiography to differentiate between external compression and other causes of stenosis like atherosclerosis. Treatment involves surgical release of the median arcuate ligament to restore celiac artery perfusion. Surgical treatment outcomes vary with the population studied but consistently display symptomatic relief in over 50% of patients. MALS is a diagnosis of exclusion that may be the cause of chronic abdominal pain. Anatomical abnormalities can be found in many individuals but investigating their roles in symptomatic etiology is challenging, as represented by this case.
